# Panel discussion on the application of MCDA tools

**DOI:** 10.1186/s12962-018-0130-y

**Published:** 2018-11-09

**Authors:** Michael Watson

**Affiliations:** Moderna Therapeutics, Cambridge, MA USA

**Keywords:** Prioritization, Healthcare, Multi-Criteria-Decision-Making-Analysis, MCDA, Health technology assessments (HTA), Ethiopia

## Abstract

Prioritization in healthcare is particularly sensitive to subjective biases and data asymmetry. Yet making data-based and objective decisions are critical to optimizing long term individual and societal benefit. Multi-Criteria-Decision-Making-Analysis (MCDA) provides shared processes, structure, and language to enable this. This panel presented and discussed three examples of MCDA application to health technology assessment, national healthcare strategy and balancing the priorities of providers and customers.

## Background

Prioritization is only required if resources are constrained and prioritization is only possible if we start with a specific perspective and set of objectives in mind. This is especially true of healthcare decision-making and prioritization. We are forced to clash social norms against market norms and multiple world views against limited fiscal space. The challenge is magnified by the constantly ticking clock of human suffering and the need to make trade-offs between human lives, knowing that in many cases the more valuable but more long-term preventative investments will be compared to less valuable but more immediately visible therapeutic interventions. Whilst maximum utility for everyone, forever, may be a dream, anything that gets us closer to a Ramseyian bliss point [1] of optimized utility for all should be welcomed. Multi-Criteria-Decision-Making-Analysis (MCDA) is intended to be a step in this utopic direction. Its goal is to put in place processes, structure, and language that allow us to more objectively define preferences in healthcare.

MCDA is designed to integrate multiple, often conflicting inputs and objectives and to translate them into common units. These in turn can be placed against a relevant, single scale of value to help us take our decisions. The process, structure and language are essential to ensuring that every aspect of MCDA is transparent and explicit, thereby minimizing interpretation and biases, intentional or not. MCDA can be applied to a range of applications including; benefit-risk assessments, health technology assessments, portfolio decision analyses and commissioning decisions and priority setting frameworks [2].

As with all innovation, the first iteration will be neither the best nor the last. But it is essential to kick start a continuous cycle of defining the problem to be solved, and then designing, prototyping, testing, modifying, retesting and remodifying solutions.

The panel discussion looked at three real-world examples of MCDA application and how MCDA can be applied and what lessons beta-testing has taught us: The first example examines the application of MCDA to health technology assessment (HTA). It shows how MCDA could improve cost-utility analysis (CUA) by adding dimensions of equity, burden of disease and broader social benefits to the quality and length of life focused QALY. It also highlights how testing of MCDA for HTA demonstrates methodological and practical challenges, especially in LMICs, and it discusses potential solutions.

The example from Ethiopia demonstrates how MCDA can be applied to multiple healthcare questions ranging from a 20-year strategy for transforming the health sector, through to a set of interventions envisaged to optimize primary health care impact, whilst minimizing out-of-pocket costs for its’ consumers.

The third case highlights how different the customers’ priorities can be from the providers’ and how MCDA offers a way to integrate this into a more holistic set of healthcare preferences but that doing so will require innovation in public opinion research to generate meaningful and accepted data.

## Conclusions

The three examples presented here confirm that Multi-Criteria-Decision-Making-Analysis (MCDA) offers a broadly applicable approach to reducing subjective biases and data asymmetry in healthcare decision making and prioritization.
